# Recent advances in the role of endogenous hydrogen sulphide in cancer cells

**DOI:** 10.1111/cpr.13449

**Published:** 2023-03-16

**Authors:** Hao‐Jie Chen, Ke Li, Yang‐Zhe Qin, Jing‐Jing Zhou, Tao Li, Lei Qian, Chang‐Yong Yang, Xin‐Ying Ji, Dong‐Dong Wu

**Affiliations:** ^1^ School of Basic Medical Sciences Henan University Kaifeng Henan 475004 China; ^2^ Henan International Joint Laboratory for Nuclear Protein Regulation Henan University Kaifeng Henan 475004 China; ^3^ School of Nursing and Health Henan University Kaifeng Henan 475004 China; ^4^ Kaifeng Key Laboratory of Infection and Biological Safety, School of Basic Medical Sciences Henan University Kaifeng Henan 475004 China; ^5^ School of Stomatology Henan University Kaifeng Henan 475004 China

## Abstract

Hydrogen sulphide (H_2_S) is a gaseous neurotransmitter that can be self‐synthesized by living organisms. With the deepening of research, the pathophysiological mechanisms of endogenous H_2_S in cancer have been increasingly elucidated: (1) promote angiogenesis, (2) stimulate cell bioenergetics, (3) promote migration and proliferation thereby invasion, (4) inhibit apoptosis and (5) activate abnormal cell cycle. However, the increasing H_2_S levels via exogenous sources show the opposite trend. This phenomenon can be explained by the bell‐shaped pharmacological model of H_2_S, that is, the production of endogenous (low concentration) H_2_S promotes tumour growth while the exogenous (high concentration) H_2_S inhibits tumour growth. Here, we review the impact of endogenous H_2_S synthesis and metabolism on tumour progression, summarize the mechanism of action of H_2_S in tumour growth, and discuss the possibility of H_2_S as a potential target for tumour treatment.

## Graphical abstract

Like Yin and Yang, hydrogen sulphide plays a dual role in tumours. Increased production at low concentrations (endogenous) promotes tumorigenesis and development, whereas supplementation at high concentrations (exogenous) suppresses tumours.

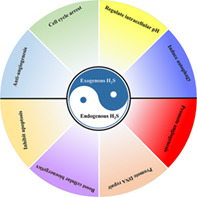

## 1 INTRODUCTION

Hydrogen sulphide (H_2_S) is one of the three known gaseous signalling molecules in biological systems. Together with carbon monoxide (CO) and nitric oxide (NO), it forms a family of endogenous gases. These gases are involved in regulating a variety of physiological and pathological processes^1^, ^2^, ^3^ and show pleiotropy and dose dependence on a variety of diseases, including cancer.^4^, ^5^, ^6^, ^7^ At present, some compounds that can inhibit or induce the synthesis of these gases have been tested in preclinical research, including NO‐releasing drugs for cancer prevention and treatment and CO‐releasing drugs for immune inflammation or autoimmune diseases.^8^, ^9^, ^10^, ^11^, ^12^, ^13^

H_2_S mainly comes from different substrates catalysed by cystathionine (CTH) β‐synthase (CBS), CTH γ‐lyase (CSE), and 3‐mercaptopyruvate sulfurtransferase (3‐MST).^14^, ^15^, ^16^ In cancer cells, H_2_S shows cytoprotective or cytotoxic effects, depending on the concentration: that is, a low concentration (endogenous) of H_2_S can induce tumorigenesis, while a high level (exogenous) of H_2_S can inhibit tumorigenesis.^17^, ^18^, ^19^ This provides two different ideas for the treatment of cancer, namely inhibiting the production of endogenous H_2_S or adding exogenous H_2_S. This review summarizes the effect of endogenous H_2_S on cancer, focuses on the impact of the change of endogenous H_2_S concentration on cancer cells, and expounds its implication on cancer treatments, hoping to provide insight for follow‐up research and drug development.

## 2 ENZYMES THAT SYNTHESIZE H_2_S

### 2.1 The distribution of CBS and its catalysed reaction

In mammals, CBS is mainly found in the liver, brain, kidney, and pancreas.^20^, ^21^ In the liver, the content of CBS is most abundant in hepatocytes and least in the hepatic stellate cells (HSCs) and Kupffer cells.^22^, ^23^ CBS is expressed in all brain regions except the hippocampus, with the highest content present in the cerebellum and cerebral cortex.^24^ CBS is also expressed in neural stem cells and regulates their differentiation.^25^ In the kidney, CBS is mainly distributed in the glomeruli, the epithelium of the proximal tubules, collecting ducts, and the inter‐lobular arteries of the kidney.^26^, ^27^ Moreover, CBS is abundantly expressed in acinar cells of the pancreas, and can also be detected in pancreatic islet cells and exocrine cells.^28^, ^29^ CBS content in other tissues is relatively low. In the digestive system, CBS exists in the gastric mucosa, colonic epithelium, small intestine, jejunum, and ileum.^30^, ^31^, ^32^, ^33^ CBS is also significantly expressed in the spleen.^34^ CBS has been suggested to play an important role in the female reproductive system since it is well expressed in the ovary and uterus but is relatively low in the prostate and testis.^35^ It is also expressed in the prostate epithelium, bladder, and urethra.^36^, ^37^, ^38^ In the heart, CBS is expressed in cardiomyocytes, coronary arteries, and perivascular adipose tissue.^39^, ^40^ Meanwhile, in the lung, it is expressed in the epithelial cells of the alveoli, bronchiole, and trachea, as well as the endothelial cells (ECs) and smooth muscle cells of the pulmonary artery.^41^, ^42^, ^43^, ^44^ In addition, the content of CBS in the thyroid is low and it is significantly increased in thyroid cancer.^45^ Likewise, CBS is not contained in breast tissue but is overexpressed in breast cancer (BC).^46^

CBS can generate H_2_S through several condensation reactions including those of two molecules of L‐cysteine into L‐lanthionine, two L‐homocysteine molecules into L‐homolanthionine, and L‐cysteine and L‐homocysteine into L‐cystathionine.^3^, ^47^ Although a large amount of cystathionine (CTH) can theoretically inhibit or even reverse the overall response of CBS, the level of CTH in most tissues is very low, so it is difficult to achieve this reverse reaction in vivo.^48^

### 2.2 The distribution of CSE and its catalysed reaction

As the main H_2_S synthase, CSE is mainly expressed in the cardiovascular and respiratory systems,^49^, ^50^ including in the liver, kidney, pancreas, uterus, and prostate.^50^, ^51^, ^52^ In addition, a small amount of CSE mRNA has also been detected in the brain, but because the inhibitor of CSE could not impede the production of H_2_S in the brain, it is thought that CSE is not the main H_2_S producing enzyme in the brain.^24^

CSE can decompose cysteine into pyruvate, ammonia, and thiocysteine, and further catalyse thiocysteine to produce H_2_S. CSE can also use homocysteine as a substrate to generate H_2_S. CSE deficiency can lead to cystathioniuria and hyperhomocysteinemia.^53^

### 2.3 The distribution of 3‐MST and its catalytic reaction

3‐MST is found in almost all tissues of mammals; however, its expression is tissue‐specific. In the central nervous system, 3‐MST is mainly located in hippocampal vertebral neurons, cerebellar Purkinje cells, and olfactory bulb mitral valve cells.^54^ In addition, 3‐MST is also relatively high in the kidney, liver, testis, large intestine, and endocrine organs.^55^

3‐MST catalyses the production of H_2_S and requires the assistance of cysteine aminotransferase (CAT).^56^ CAT converts cysteine to 3‐mercaptopyruvate, and 3‐MST transfers sulphur from 3‐mercaptopyruvate to sulphite, sulphur acceptor, or sulphur. However, this method can only generate sulphane sulphur or combined sulphur, but for producing H_2_S, the action of reducing agents (e.g., thioredoxin, dihydrolipoic acid) or various enzymes in the cell is required.^57^, ^58^

## 3 THE TUMOUR‐PROMOTING MECHANISM OF H_2_S

### 3.1 H_2_S promotes angiogenesis

Angiogenesis is a multi‐step process involving ECs that is characterized by endothelial extracellular matrix remodelling, including initiation, migration, catheter formation, and differentiation.^59^ When gene mutations accumulate and cause cancer, the solid tumour will form a highly vascularized state. These vessels provide oxygen and nutrition for the development or local spread of the tumour.^60^

H_2_S promotes EC angiogenesis by regulating cyclic nucleotides, kinases, and ion channels.^61^, ^62^ H_2_S donors increase the phosphorylation levels of Akt, p38, and ERK1/2, while the pharmacological inhibition of PI3K/Akt and MAPK inhibits the proliferation and migration of EC. H_2_S also promotes angiogenesis through the K_ATP_ channel. In addition, in human EC, the K_ATP_ channel plays a role upstream of p38.^63^, ^64^, ^65^, ^66^, ^67^ The inhibition of endothelial nitric oxide synthase, soluble guanylyl cyclase, or cyclic guanosine monophosphate (cGMP) dependent protein kinase weakens H_2_S‐stimulated angiogenesis, indicating H_2_S can interact with multiple molecules of the NO/cGMP pathway to promote angiogenesis.^68^, ^69^

Vascular endothelial growth factor (VEGF) can promote vascular permeability, extracellular matrix degeneration, vascular EC migration, proliferation, and angioplasty.^70^, ^71^ Many studies have shown that there is extensive interaction between H_2_S and VEGF. In particular, the incubation of human EC with VEGF increases the concentration of H_2_S, and the silencing or pharmacological inhibition of CSE weakens the angiogenesis of VEGF‐stimulated EC.^66^ Although the mechanism of this phenomenon has not been clarified, some experiments show that it may be caused by CSE‐mediated Ca^2+^/calmodulin‐dependent activation.^69^ In addition, the inhibition of CSE can markedly block the activation of p38 and ERK1/2 stimulated by VEGF.^66^ CBS silencing also reduced the expression of VEGFR2 and neuropilin‐1, thereby reducing the signal intensity of VEGF. The S‐sulfhydration of specificity protein 1 (Sp1) at Cys68 and Cys755 by H_2_S enhances the stability of Sp1, and subsequently, promotes the transcription of VEGFR2.^72^ H_2_S can also enhance binding of VEGF to VEGFR2 (thereby increasing activity of the latter).^73^

### 3.2 H_2_S inhibits apoptosis

Apoptosis refers to the autonomous and orderly death of cells controlled by genes to maintain the stability of the internal environment. It involves the activation, expression, and regulation of a series of genes.^74^ Evasion of apoptosis is an important mechanism in the development of cancer, allowing cancer cells to survive under physiological stress.^75^ H_2_S has been found to play an anti‐apoptotic effect in the cardiovascular system, ischaemia–reperfusion injury, and various cancers.^76^, ^77^, ^78^, ^79^, ^80^ One of the potential anti‐apoptotic mechanisms of H_2_S is its anti‐oxidant effect achieved by scavenging reactive oxygen species (ROS) and reactive nitrogen species (RNS). Although H_2_S is usually at a low concentration under baseline conditions, its small molecular structure and ability to penetrate freely on the cell membrane make it a more effective antioxidant than glutathione (GSH). However, it is reasonable to believe that H_2_S‐mediated antioxidant protection is caused by a wide range of intermediate signals it regulates rather than direct ROS/RNS clearance.^81^ Another potential mechanism is the activation of anti‐apoptotic pathways via S‐sulfhydrating NF‐κB, Kelch‐like ECH‐associated protein 1, and mitogen‐activated protein kinase kinase 1 (MEK1).^82^, ^83^, ^84^

### 3.3 H_2_S boosts cellular bioenergetics

Cellular bioenergetics plays an important role in the occurrence and development of different types of cancer.^85^, ^86^ Initially, H_2_S was reported to exhibit cytotoxic effects on mitochondria by inhibiting the cytochrome c oxidase system, but recent studies demonstrate a more complex, concentration‐dependent regulation of mitochondrial and cellular bioenergetics by H_2_S. In normal intestinal epithelial cells, H_2_S acts as a substrate for bioenergy production.^87^, ^88^ Further research shows that in colon and ovarian cancer, H_2_S can serve both as a regulator and a substrate of bioenergetics.^89^, ^90^ CBS silencing reduces oxygen consumption and adenosine triphosphate (ATP) production. Silencing of 3‐MST in hepatoma cells also shows similar effects. Likewise, the pharmacological inhibition of CBS and 3‐MST blocks electron transport and mitochondrial energy production in various cancer cells, whereas replenishment of substrates for these enzymes reversed this process.^91^, ^92^, ^93^, ^94^, ^95^ It is worth adding that, H_2_S by itself cannot initiate or maintain the mitochondrial electron transport system, but can affect glycolysis‐derived electron donors.

The H_2_S‐mediated mitochondrial electron transport requires the participation of sulphide quinone oxidoreductase (SQR),^87^, ^96^, ^97^ and the expression of SQR in tumour cells is up‐regulated under hypoxic conditions that may be a potential mechanism for tumour cells to use H_2_S to generate energy.^98^ On the other hand, electrons from SQR can also be transported in reverse when cells are exposed to higher concentrations of H_2_S.^87^ In cancer cells, this mechanism does not aid in electron transport, proton pump, or ATP generation, but instead it stimulates mitochondrial ROS production.^99^ In addition, H_2_S can directly S‐sulfhydrate glyceraldehyde‐3‐phosphate dehydrogenase (GAPDH) to enhance its activity in ATP generation.^52^

### 3.4 H_2_S promotes DNA repair and tumour growth

Recent studies have shown that cell cycle checkpoints, DNA damage and repair, and the expression of proteins involved in maintaining gene stability are regulated by both exogenous and endogenous H_2_S.^100^, ^101^, ^102^ The effect is suggested to be due to be associated with activities of MEK1 and poly [ADP‐ribose] polymerase 1 (PARP‐1). Specifically, PARP1 can sense DNA single‐strand or double‐strand breaks and initiate DNA damage repair pathways. PARP inhibitors have been developed to block DNA repair in BRCA‐mutated cancers, thereby initiating signalling pathways that trigger apoptosis and ultimately inhibit tumour growth.^103^ H_2_S is abnormally elevated in a variety of cancers, and inhibition of CBS or CSE activity suppresses tumour growth in colon, lung, prostate, and BCs.^89^, ^93^, ^104^, ^105^ MEK1 belongs to the classical MAPK kinase pathway, and the activation of MEK1 is closely related to cell proliferation and tumorigenesis.^106^ S‐sulfhydration of MEK1 by H_2_S at Cys341 promotes phosphorylation and nuclear translocation of MEK1, thereby activating PARP‐1‐mediated DNA damage repair, which is most likely a key driver of tumour growth due to CBS or CSE overexpression.^84^ In addition, H_2_S can S‐sulfhydrate Exo/endonuclease G at Cys76 in mitochondria to mediate DNA damage repair in mitochondria.^107^

## 4 ENDOGENOUS H_2_S, CELL SIGNAL TRANSDUCTION, AND CANCER

### 4.1 Endogenous H_2_S and BC

BC is the most common malignancy in women and is divided into different subtypes with widely varying prognoses and treatment modalities.^108^, ^109^ Endocrine therapy is suitable for hormone receptor (HR)‐positive patients, and targeted therapy is suitable for human epidermal growth factor receptor 2 (HER2)‐positive patients.^110^ Triple‐negative breast cancer (TNBC) is a highly aggressive subtype lacking oestrogen receptor, progesterone receptor, and HER2, and it is easy to metastasize to the nervous system and lungs.^111^, ^112^, ^113^

CBS and CSE have been shown to be highly expressed in various BC types and are closely related to their development. In HR^+^/HER2^+^ BC cell line MCF7, knockdown of CBS and CSE inhibited cell growth by inhibiting the Akt signalling pathway, while in TNBC cell line MDA‐MB‐231, knockdown of CBS inhibited cell growth by inhibiting signal transducer and activator of transcription 3 (STAT3).^114^ NO regulates the growth of various tumours and has a positive feedback loop with H_2_S.^115^ Knockdown of CBS and CSE mitigates the production of NO, while the addition of NO donors attenuates the antitumor effect of CBS and CSE knockdown.^114^ CSE overexpression also promotes the metastasis of BC, especially in TNBC. In vitro experiments have shown that CSE promotes the growth, migration, and invasion of BC. Nude mice experiments show that CSE promotes the migration of BC cells to the lungs, which may be related to the elevated expression of matrix metallopeptidase (MMP)‐2 and MMP‐9. In addition, knockdown of CBS and CSE inhibits the PI3K‐Akt (PI3K, Akt, and pAkt), focal adhesion kinase‐paxillin, and Ras‐MAPK (Ras, Raf, ERK1/2, and pERK1/2) pathways,^116^ thus confirming the promoting effect of CBS and CSE on BC. CBS is also highly expressed in basal‐like breast cancer (BLBC). Protein‐Cysteine persulfidation by CBS causes an increase in GSH synthesis. Silencing CBS increases the expression of angiogenesis inhibitor SERPINF1 and inhibits the expression of Ki67, CD31, CD34, and hypoxia‐inducible factor (HIF‐1α), and reduces GSH synthesis enhanced oxidative stress, thereby inhibiting tumour cell growth. CSE abolishes the tumour suppressor effect due to CBS knockdown to a certain extent, indicating that simultaneous targeting of CBS and CSE can produce a more obvious inhibitory effect on BLBC.^117^ STAT3 is a transcription factor that is highly activated in BC and promotes the growth of cancer cells.^118^ STAT3 can directly regulate the expression of CSE. At the same time, STAT3 is also regulated by CSE and is positively correlated with the expression of CSE. Knocking down CSE significantly reduces proliferation and migration activities in BC cells.^105^ In addition, CBS and CSE also regulate the immunogenicity of BC cells. After silencing CBS and CSE, the expression of Natural Killer Group 2D ligands (ULBP2 and MICA) increases, which improves the targeting of NK cells to BC cells. At the same time, the expression of co‐stimulatory ligands CD86 and 41BBL on BC cells increased, and these ligands bind to homologous receptors CD28 and 41BB on T cells to activate T cells and enhance their function. Tumour necrosis factor α (TNF‐α) promotes immune cell apoptosis in the tumour microenvironment, and TNF‐α expression is also reduced after CBS and CSE knockdown.^114^ In addition, the level of reactive aldehyde (such as 4‐hydroxynonenal and malondialdehyde) adducts in BC cells co‐cultured with macrophages increases after CBS silencing, resulting in cytotoxicity.^119^ The recently discovered novel CSE inhibitor I157172 up‐regulates sirtuin 1 (SIRT1) and inhibits the phosphorylation and deacetylation of STAT3, the expression of MMP2/9, p‐Akt, and Bcl‐2, which in turn inhibits the migration and invasion of BC cell line MCF7.^120^

**FIGURE 1:**
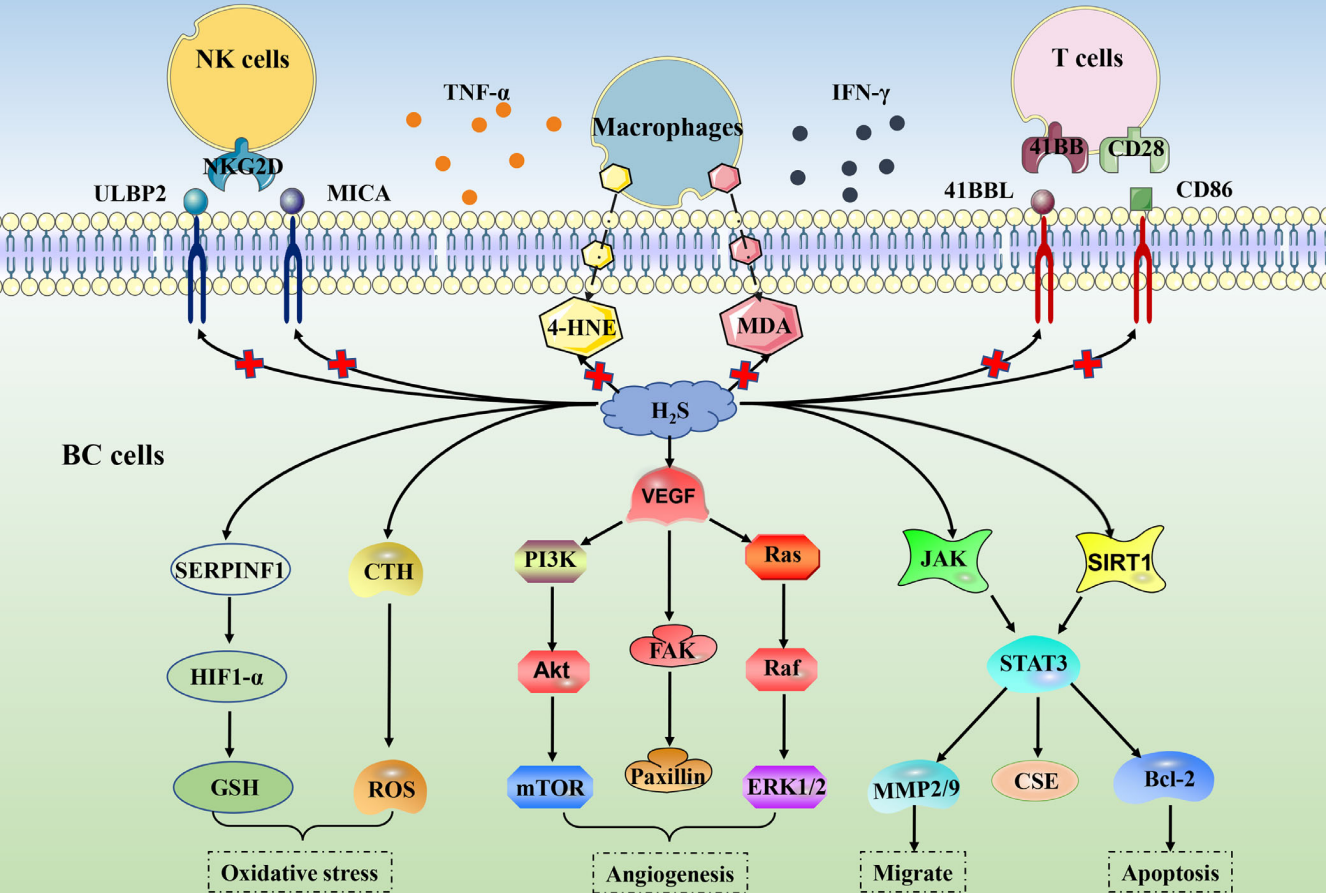
Hydrogen sulphide (H_2_S) can regulate the occurrence and development of breast cancer cells by regulating oxidative stress, angiogenesis, migration, apoptosis, and immunogenicity. 4‐HNE, 4‐hydroxynonenal; CD80, CD86, 41BBL, T cells co‐stimulate the ligand; CSE, cystathionine γ‐lyase; CTH, cystathionine; FAK, focal adhesion kinase; GSH, glutathione; HIF1‐α, hypoxia‐inducible factor; JAK, Janus kinase; MDA, malondialdehyde; MMP, matrix metallopeptidase; NKG2D, Natural Killer Group 2D; ROS, reactive oxygen species; SERPINF1, angiogenesis inhibitor; SIRT1, sirtuin 1; STAT3, signal transducer and activator of transcription 3; ULBP2 and MICA, NKG2D ligands; VEGF, vascular endothelial growth factor;

In addition, CTH, an intermediate metabolite of the CBS‐catalysed synthesis of H_2_S, has recently been found to exert anti‐apoptotic effects in cells.^121^ Due to the elevated levels of CBS in BC, the production of CTH intensifies, but its downstream metabolite CGL remains insignificantly affected, resulting in the accumulation of CTH in BC cells. Exogenously added CTH attenuates H_2_O_2_‐induced and doxorubicin‐induced apoptosis and maintains mitochondrial stability by increasing intra‐cytoplasmic calcium concentration, restoring the number of mitochondrial cristae, and increasing mitochondrial reserve and exerting anti‐apoptotic effects in BC cells^46^ (Figure 1).

### 4.2 Endogenous H_2_S and hepatoma

The role of endogenous H_2_S in hepatoma was shown to be twofold, which appears to be related to the cell type and the reaction mechanism of H_2_S synthase. In addition, a variety of factors also affect hepatoma by regulating endogenous H_2_S. CSE is highly expressed in hepatoma cell lines HepG2 and PLC/PRF/5, but low in Hep3B, and its silencing shows an inhibitory effect in HepG2 and PLC/PRF/5, while the effect on Hep3B was not obvious. Subsequent experiments showed that the tumour suppressor effect caused by the knockdown of CSE was achieved by regulating apoptotic proteins (p53, Bax, Bcl‐2, p21, and caspase‐3), key proteins of EGFR and MAPK signalling pathways, and increasing the production of ROS to induce RNA damage.^122^ High expression and over‐activation of indoleamine 2,3‐dioxygenase 1 (IDO1) are important reasons for the immune evasion of cancer cells.^123^, ^124^ In hepatocellular carcinoma (HCC) patients, the expression of IDO1 is negatively correlated with the expression of CSE. The deletion of H_2_S in CSE^−/−^ mice leads to the increased expression and activity of IDO1. Exogenously added H_2_S down‐regulates the expression of IDO1 through the NF‐κB and STAT3 pathways and inhibits the activity of IDO1 through the nitric oxide synthase/NO pathway. In addition, exogenously added H_2_S also inhibited tumour growth in H22 hepatoma mice by inducing effector T cells and suppressing myeloid‐derived suppressor cells.^125^ The effect of other factors on cancer may also play a role through the CSE/H_2_S axis. Irradiation increased the long‐term migration and invasion ability of HepG2 cells. This effect was due to the increased expression of CBS and CSE caused by radiation, and the activation of epithelial–mesenchymal transition (EMT) and P38/MAPK signalling pathways. After knocking down CBS and CSE, the p38/EMT signalling pathway was inhibited, and the effect was more obvious after knocking down CSE.^126^ The PI3K/Akt signalling pathway plays a role in promoting invasive phenotype, malignancy, angiogenesis, and so forth in a variety of cancers.^127^, ^128^ The activated PI3K /Akt pathway promotes the occurrence and development of HCC through Sp1‐mediated regulation of CSE promoter and protein expression, indicating a positive feedback loop between CSE and PI3K/Akt.^129^ CBS exhibits different roles in different hepatoma cell lines. In HCC cell lines Hep3B and MHCC97H, CBS inhibits tumour growth by regulating apoptosis‐related proteins (cleaved Caspase‐3 and Bcl‐3) and inhibiting the transcription of paired related homeobox 2 by interleukin‐ 6 (IL‐6), which negatively regulates the expression of STAT3. Regulatory T cells are the main inhibitory component of the immune system and are controlled by forkhead box P3 (FOXP3). In regulatory T cells, the absence of CBS leads to the activation of IL‐6/STAT3 and promotes the expression of FOXP3, which activates regulatory T cells and suppresses T cells to help HCC cells evade immune attack.^130^ But in the human hepatoma cell line HepG2, inhibiting CBS results in cancer suppression. The combined application of curcumenol and laminarin inhibited the proliferation and metastasis of HepG2 cells. Subsequent experiments found that this effect was caused by attenuating the expression of CBS as well as STAT3 (pSTAT3), Bcl‐2, MMP2, MMP9, VEGF, and their downstream signalling pathways (ERK1/2, pERK1/2, Akt, pAkt).^131^ 3‐MST inhibited the Akt/FOXO3a/p27 and cyclin D1/CDK4/Rb/E2F1 signalling pathways by increasing the production of H_2_S and inhibited the proliferation, migration, and invasion of HCC cell lines HepG2 and MHCC‐LM3 by increasing the cleaved caspase‐3 and PARP levels.^132^

**FIGURE 2:**
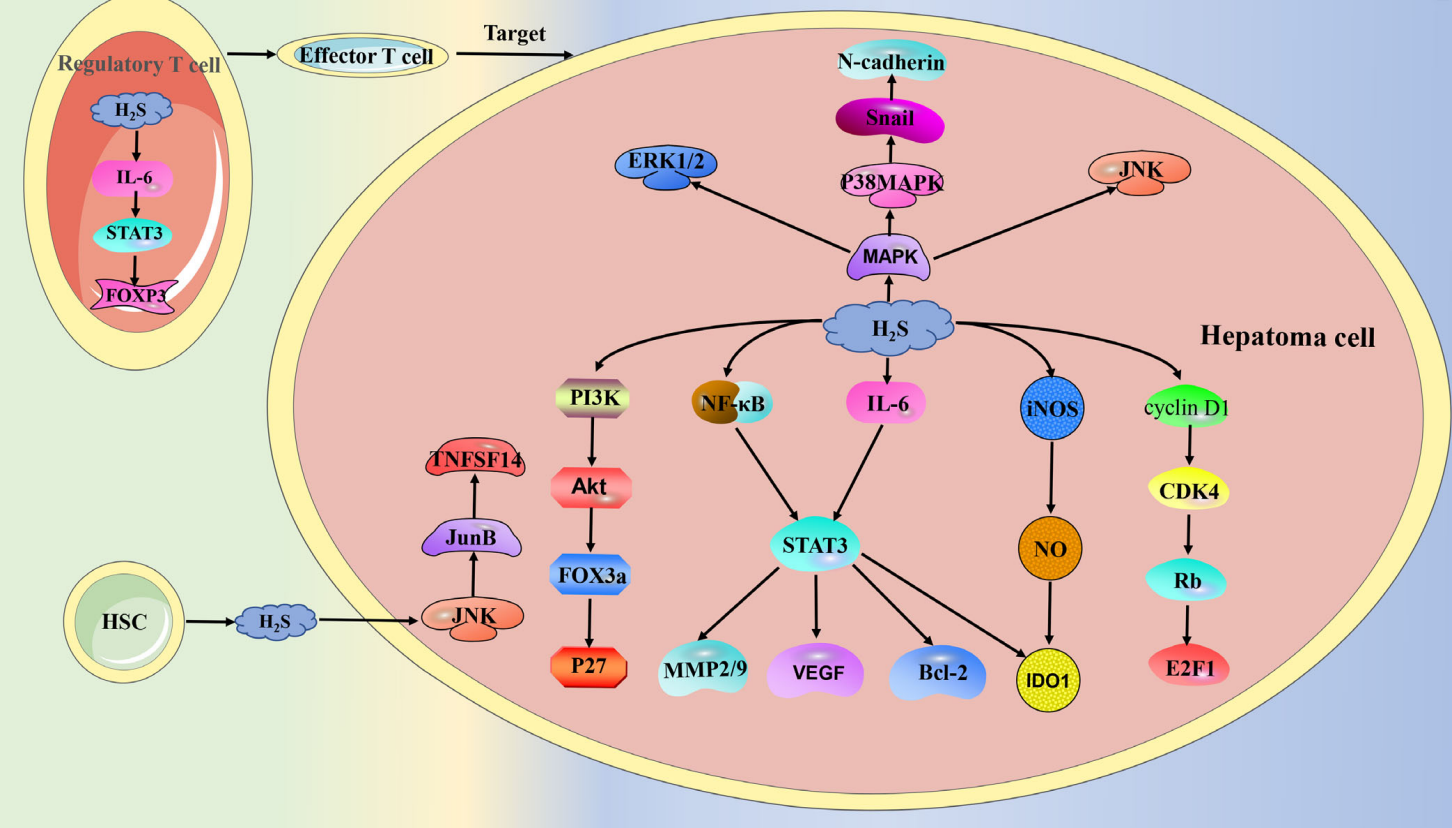
In addition to the hydrogen sulphide (H_2_S) synthesized by the hepatoma cells themselves, the H_2_S secreted by the hepatic stellate cells also has an effect on the hepatoma cells. CDK4, recombinant cyclin dependent Kinase 4; E2F1, E2F transcription factor 1; FOX3a, forkhead box O3; FOXP3, forkhead box P3; HSC, hepatic stellate cell; IDO1, indoleamine 2, 3 ‐dioxygenase 1; IL‐6, interleukin‐ 6; iNOS, nitric oxide synthase; JNK, c‐Jun N‐terminal kinase; JunB, JunB Proto‐Oncogene; MMP, matrix metallopeptidase; NF‐κB, nuclear factor kappa‐B; NO, nitric oxide; P27, CDK inhibitor P27; Rb, retinoblastoma; STAT3, signal transducer and activator of transcription 3; TNFSF14, pro‐apoptotic factor; VEGF, vascular endothelial growth factor

Other cells in the liver can also inhibit the occurrence and development of HCC by secreting H_2_S. HSCs can play a role in suppressing tumours in the development of HCC. Activated HSCs release H_2_S and H_2_S increases the pro‐apoptotic factor TNFSF14 through the JNK/JunB signalling pathway^133^ (Figure 2).

### 4.3 Endogenous H_2_S and colorectal cancer

CBS shows different effects in different colorectal cancer cell lines. In HT‐29 cells, high expression of CBS inhibits cell proliferation, clone formation, spheroid formation, migration, cell growth, and liver metastasis in vitro and in vivo by inhibiting transcription factor Sp1 and down‐regulating CD44 (a transmembrane glycoprotein and an important biomarker of cancer stem cells) expression.^134^ In SW480 and DLD1 cells, there is a positive feedback regulation between CBS and VEGF. Knockdown of CBS diminishes the expression of VEGF by regulating activating protein 1, while bevacizumab (anti‐VEGF monoclonal antibody) reduces the binding of NF‐κB to the CBS promoter and inhibits CBS gene activation. Moreover, CBS knockdown reduces colon cancer migration, invasion, and angiogenesis by inhibiting VEGF.^135^ In HCT116 and HT29 cells, aminooxyacetic acid (AOAA) hinders cell survival and intracellular H_2_S synthesis in a concentration‐dependent manner. Furthermore, the combined application of AOAA and oxaliplatin (OXA)‐enhanced OXA‐induced apoptosis by modulating the level of apoptotic markers (up‐regulate cleaved caspase‐9, cleaved PARP, Bax, and p53 and down‐regulate Bcl‐2, total caspase‐9, total caspase‐3, and total PARP), inducing the production of ROS, and reducing the generation of intracellular GSH in vitro, while in vivo AOAA and OXA decreased the expression of Ki67 and proliferating cell nuclear antigen (PCNA) thus enhancing the chemotherapeutic effect of OXA.^136^ AOAA also enhances the sensitivity of colon cancer cells to 5‐Fluorouracil (5‐FU) Combination treatment of AOAA and 5‐FU‐induced apoptosis, cell cycle arrest, disturbance of bioenergetic production, and increased oxidative stress. MiR‐215‐5p is a key tumour suppressor in colon cancer, AOAA and sh‐CBS can both increase the expression of miR‐215‐5p and decrease the expression of epiregulin and thymidylate synthetase, and enhance the sensitivity of acquired 5‐FU‐resistant cell lines to 5‐FU.^137^ In HCT116 and NCM356 of colon cancer cells, sh‐CBS and AOAA mitigate basal respiration, ATP synthesis, and glycolysis by inhibiting GAPDH and L‐cysteine.^89^ N1, N12‐Diacetylspermine can up‐regulate the expression of CBS and promote the proliferation of colorectal cancer cell lines SW480 and Caco2, while miR‐559 can target and inhibit CBS and inhibit the proliferation of the cells.^138^ These opposite results of CBS may be caused by the difference in cell lines, and AOAA, as a pyridoxal phosphate‐dependent enzymes inhibitor, showed an inhibitory effect on both CBS and CSE.^139^, ^140^ 3‐MST is highly expressed in murine colon cancer cell line CT26. HMPSNE (a 3‐MST inhibitor) inhibited the growth, migration, and viability of CT26 cells by inhibiting the cell metabolic capacity and glycolysis parameters but had no obvious effect on cell apoptosis and necrosis.^93^ Furthermore, in HCT116 cells, AOAA and HMPSNE induced mesenchymal‐epithelial transition of cells via transcription factor Sp3‐ATP citrate lyase‐Wnt‐β‐Catenin. *N*‐acetylcysteine (NAC) is a widely used antioxidant that acts as a substrate for 3‐MST with higher efficiency than cysteine, but its role in cancer is bidirectional.^141^, ^142^ In SW480 cells, exogenous supplementation of NAC increased the expression and activity of 3‐MST and SQR. Since 3‐MST exists as a cancer promoter in colorectal cancer, it seems that NAC can lead to the development of colorectal cancer, but whether it can lead to drug resistance in colorectal cancer cells by regulating H_2_S and oxidative stress needs further evaluation.^143^ When converted to cysteine, xCT (also known as SLC7A11) acts as a precursor for GSH biosynthesis, and increased xCT expression is associated with chemoresistance and nutrient dependence in a variety of cancers.^144^, ^145^ In human colon cancer cells HCT116 and HT29, xCT is highly expressed, and CES‐derived H_2_S S‐sulfhydrates OTU domain‐containing ubiquitin aldehyde‐binding protein 1 (OTUB1) at Cys91 to regulate its binding to xCT. Inhibition of CSE attenuates the S‐sulfhydration of OTUB1 and reduces xCT production. In addition, the production of GSH and H_2_S was reduced after the inhibition of xCT and CSE, which may lead to cell oxidative stress‐induced apoptosis. In vivo experiments also showed that after the knockdown of CSE and xCT, the expression of PCNA was decreased, and the expression of prostaglandin‐endoperoxide synthase was increased, which significantly inhibited tumour growth.^146^

**FIGURE 3:**
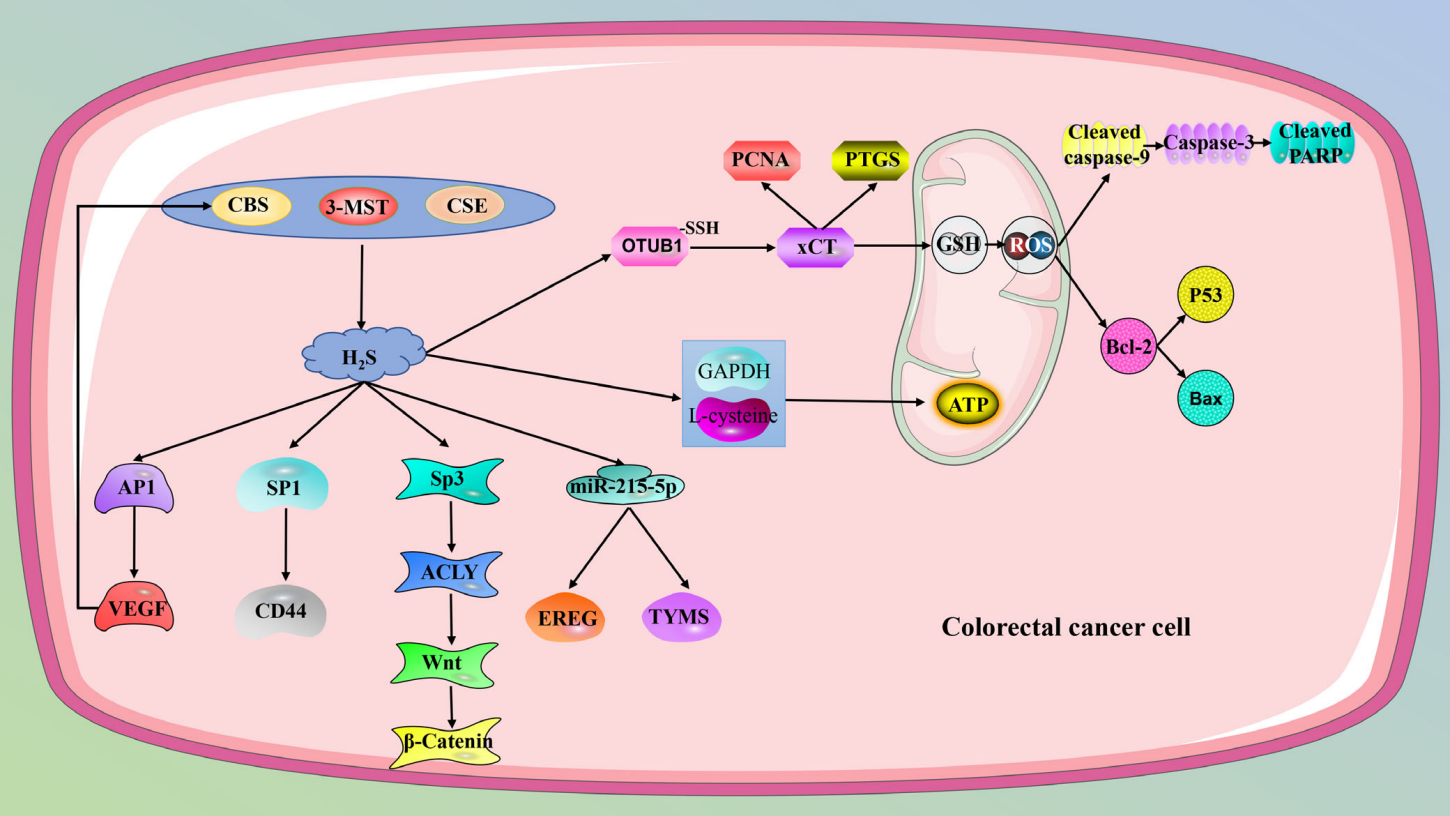
Hydrogen sulphide (H_2_S) synthase has different functions in colorectal cancer, which may be due to the crosstalk between different molecules and the high concentration of H_2_S in the intestinal environment. However, in general, inhibition of endogenous H_2_S can inhibit tumour growth. 3‐MST, 3‐mercaptopyruvate sulfurtransferase; ACLY, ATP citrate lyase; AP1, activating protein 1; ATP, adenosine triphosphate; CBS, cystathionine β‐synthase; CD44, a transmembrane glycoprotein and an important biomarker of cancer stem cells; CSE, cystathionine γ‐lyase; EREG, epiregulin; GAPDH, glyceraldehyde‐3‐phosphate dehydrogenase; GSH, glutathione; OTUB1, OTU domain‐containing ubiquitin aldehyde‐binding protein 1; PCNA, proliferating cell nuclear antigen; PTGS, prostaglandin‐endoperoxide synthase; ROS, reactive oxygen species; Sp1, specificity protein 1; Sp3, transcription factor Sp3; ‐SSH, S‐sulfhydration; TYMS, thymidylate synthetase; VEGF, vascular endothelial growth factor; xCT, SLC7A11.

Microorganisms in the gut environment can degrade cysteine to generate H_2_S, resulting in high levels of H_2_S in the gut environment.^147^ There may be some crosstalk between these H_2_S and intracellular H_2_S so that the three H_2_S‐producing enzymes exhibit such a complex and biphasic role in colorectal cancer (Figure 3).

### 4.4 Endogenous H_2_S and gastric cancer

**FIGURE 4:**
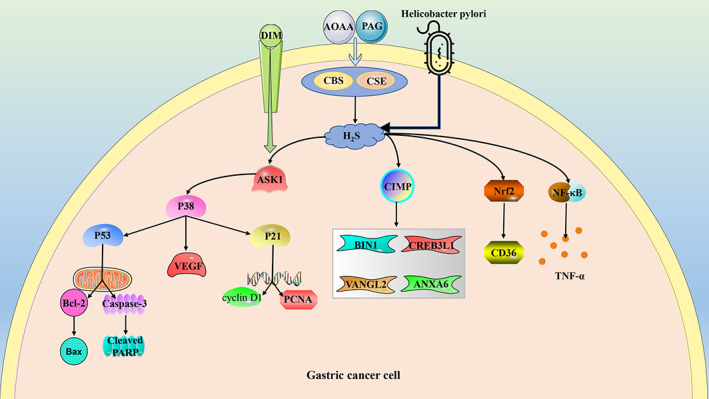
The role of hydrogen sulphide (H_2_S) in gastric cancer is a multifactorial process. ANXA6, annexin A6; AOAA, aminooxyacetic acid; ASK1, apoptosis signal‐regulating kinase 1; BIN1, bridging integrator 1; CBS, cystathionine β‐synthase; CD36, fatty‐acid receptor CD36; CIMP, CpG island methylator phenotype; CREB3L1, cAMP responsive element binding protein 3 like 1; CSE, cystathionine γ‐lyase; DIM, 3,3′‐Diindolylmethane; NF‐κB, nuclear factor kappa‐B; Nrf2, nuclear factor erythroid 2‐related factor 2; PAG, DL‐Propargylglycine; PCNA, proliferating cell nuclear antigen; TNF‐α, tumour necrosis factor α; VANGL2, VANGL planar cell polarity protein 2; VEGF, vascular endothelial growth factor.

Gastric cancer (GC) is one of the leading causes of cancer death worldwide, and the CpG Island methylator phenotype (CIMP) is an epigenetic molecular subtype that suppresses the expression of tumour suppressors in a variety of malignancies.^148^ Novel associations between CBS epimutations and CIMP subtypes in GC, marked reduction of CBS staining in malignant gastric epithelium, and in vitro models of CBS deficiency can lead to aberrant DNA methylation, down‐regulation of subsets of genes involved in tumour suppressor activity, including annexin A6, VANGL planar cell polarity protein 2, bridging integrator 1, and cAMP responsive element binding protein 3 like 1. Deletion of CBS also leads to regulation of inflammation and H_2_S production, particularly, through the elevation of TNF‐α and NF‐κB activity, which is accompanied by the reduction of H_2_S. In addition, epimutation of CBS has also been associated with CIMP in bladder urothelial carcinoma, oesophageal adenocarcinoma, head and neck squamous cell carcinoma, HCC, and uterine corpus endometrial carcinoma.^15^ CSE is highly expressed in GC cell line AGS and its inhibition by DL‐propargylglycine (PAG) and β‐cyano‐L‐alanine (BCA) markedly lowers the proliferation ability of AGS and promotes apoptosis.^149^ AOAA or PAG also enhances the sensitivity of GC cells to 3,3′‐diindolylmethane (DIM). In BGC‐823 and SGC‐7901 cells, AOAA or PAG was shown to augment the sensitivity of GC cells to DIM by activating the p38‐p53 signalling pathway and regulating downstream signalling molecules. AOAA and PAG combined with DIM suppresses cell proliferation by regulating Cyclin D1, PCNA, and p21, cell migration by impeding VEGF, and facilitates cell apoptosis by regulating cleaved PARP, cleaved caspase‐3, Bax, and Bcl‐2.^150^ In addition, fatty‐acid receptor CD36‐dependent lipid metabolism is an important component of metabolic reprogramming in cancer cells.^151^ In GC cells, overexpression of CD36 induces lipid metabolism reprogramming and promotes GC metastasis, and endogenous H_2_S mediates CD36‐induced resistance to antiangiogenic drugs and up‐regulated CD36 expression by inducing nuclear translocation of antioxidant transcriptional factor Nrf2.^152^ *Helicobacter pylori* infection induces peptic ulcer and GC, and H_2_S production is increased in *H. pylori*‐infected AGS cells, suggesting that H_2_S may be involved in *H. pylori*‐induced gastric mucosal disease^153^ (Figure 4).

### 4.5 Endogenous H_2_S and ovarian cancer

**FIGURE 5:**
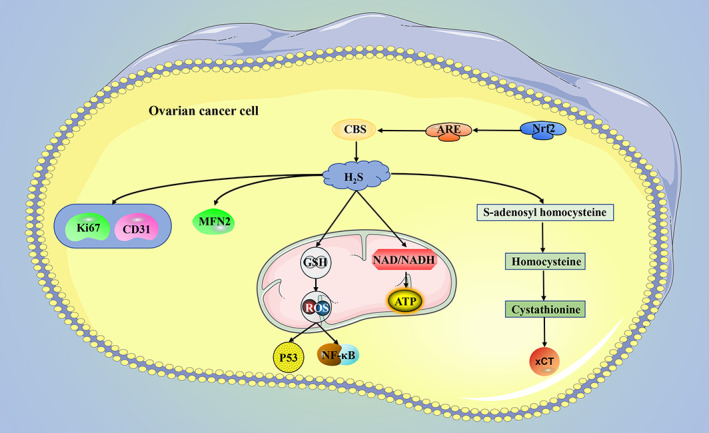
The main hydrogen sulphide (H_2_S) producing enzyme in ovarian cancer is CBS. In ovarian cancer, inhibition of CBS increases cancer cell apoptosis and ferroptosis and decreases ATP production. ARE, antioxidant response element; ATP, adenosine triphosphate; CBS, cystathionine β‐synthase; CD31, Platelet endothelial cell adhesion molecule‐1; GSH, glutathione; MFN2, mitomycin 2; Nrf2, nuclear factor erythroid 2‐related factor 2; ROS, reactive oxygen species; xCT, SLC7A11.

The synthesis of H_2_S in ovarian cancer is mainly regulated by CBS and CBS is highly expressed in both primary epithelial ovarian cancer and ovarian cancer cell lines, and down‐regulation of CBS in vitro induces oxidative stress to trigger apoptosis cascade by regulating GSH, ROS, p53, and NF‐κB in ovarian cancer cells, and reduces NAD/NADH ratio and ATP production by inhibiting mitochondrial respiration. In vivo CBS silencing inhibits tumour angiogenesis by reducing Ki67 and CD31. Meanwhile, down‐regulation of CBS enhances ovarian cancer sensitivity to cisplatin both in vivo and in vitro.^90^ Mitomycin 2 (MFN2) plays an important role in cell proliferation and death.^154^ High expression of CBS and MFN2 has a poor prognosis for ovarian cancer. Metabolites GSH and H_2_S of CBS can increase the expression of MFN2.^155^ Also, Nrf2 enhances the expression of CBS through antioxidant response element (ARE) and high expression of CBS mitigates ferroptosis induced by erastin (xCT‐specific inhibitors) in ovarian cancer by regulating S‐adenosyl homocysteine, homocysteine, and CTH.^156^ In addition, inhibition of CBS‐induced and CSE‐induced cell death in ES2 cell line.^157^ Selenium‐containing chrysin can inhibit cancer by inhibiting CBS^158^ (Figure 5).

### 4.6 Endogenous H_2_S and prostate cancer

The presence of 3‐MST was not detected in prostate cancer tissues.^159^ However, the expression of the other two H_2_S synthases is also influenced by a variety of factors. Studies have shown that CBS is not detected in benign prostatic epithelium, while low and high levels of CBS are detected in benign hyperplasia prostate cell lines and androgen‐dependent prostate cancer cell lines (LNCaP and DU145), respectively, which seems to indicate that high expression of CBS promotes the progression of prostate cancer, while low expression of CBS is found in bone metastatic cell lines of prostate cancer PC‐3. This may also be due to the fact that the expression of CBS is regulated by androgens, but the results of this regulation are contradictory: in LNCaP cells, dihydrotestosterone up‐regulates the expression of CBS while testosterone down‐regulates the expression of CBS.^36^, ^160^ CSE is highly expressed in both PC tissues and cells, but its amount varies in different types of PC cells. The expression of CSE in bone metastatic PC‐3 cell lines was higher than that in tumour‐derived PC‐3 cells, which may mean that the high expression of CSE accelerated the metastasis of PC.^104^ Experiments show that in the bone metastatic prostate cancer cell line PC‐3, CSE‐derived H_2_S leads to increased IL‐1β production through S‐sulfhydration of NF‐κB at Cys38S and increases the expression of downstream MMP‐13 and VEGF to increase cell migration and invasion. In the mouse in‐situ transplantation model, CSE knockdown inhibits tumour growth by inhibiting CD31 and vessel endothelial hyaluronan receptor 1 expression and reduces the incidence of para‐aortic lymph nodes and bone metastases.^104^ In the androgen‐resistant prostate cancer cell line LNCap, CSE‐derived H_2_S inhibits the growth of prostate cancer cells by inhibiting the trans‐activation of androgen receptor through S‐sulfhydrate at Cys611/614.^161^ However, it has been reported that H_2_S can increase LNCap mitosis by activating T‐type calcium channels,^162^ and dihydrotestosterone can down‐regulate CBS and CSE expression.^36^ In general, H_2_S plays a different role in prostate cancer by regulating different molecules.

### 4.7 Endogenous H_2_S and other cancers

Endogenous H_2_S also has different effects on other types of cancer. In non‐small cell lung cancer (NSCLC) cell lines A549 and 95D, the expressions of CBS and CSE were significantly up‐regulated. AOAA and PAG inhibited the growth of NSCLC by regulating the expressions of cleaved caspase‐3, Bax, and Bcl‐2, and inhibited the growth of NSCLC by regulating the expressions of E‐cadherin, vimentin, MMP2, and MMP9. Silencing of CBS or CSE shows the same tumour‐suppressing effect. HIF‐1α is critical for H_2_S‐mediated EMT and angiogenesis, and up‐regulation of HIF‐1α resulted in up‐regulation of CBS and CSE expression followed by increased production of VEGF, PI3K, and p‐PI3K, which was reversed by AOAA and PAG.^163^ Ribosomal proteins (rp) play a key role in the therapeutic effects of 5‐FU on tumours, and rpL3 is a key sensing molecule for 5‐FU and oxaliplatin‐induced ribosomal stress in colon and lung cancers. In lung cancer tissues, the expression of rpL3 was down‐regulated and the expression of CBS was up‐regulated. In the lung cancer cell line Calu‐6 after 5‐FU treatment, rpL3 decreased its stability at transcriptional and post‐translational levels by targeting CBS to enhance the effect of 5‐FU on cancer cells' lethality.^164^ In lung cancer cell lines A549 and H1944, high levels of H_2_S enhance the activity of mitochondrial DNA repair enzymes and improves ATP production in cancer cells, and AOAA reverses this effect and increases the sensitivity of tumour‐bearing mice to chemotherapeutics.^107^ CBS expression is also elevated in chronic myeloid leukaemia, and knockdown of CBS or AOAA treatment promotes apoptosis and triggers S‐phase arrest by regulating cleaved caspase‐9, Bax, cyt C, and NF‐κB. The high expression of CBS boosts the proliferation, migration, and invasion of oesophageal squamous cell carcinoma in vitro and induces angiogenesis and lymphatic metastasis in vivo by up‐regulating VEGF and activating the SIRT1 signalling pathway. These effects could be reversed by the knockdown of CBS. In clear cell renal cell carcinoma, either hydroxylamine (HA; a dual inhibitor of CBS and CSE) or PAG exerts tumour‐suppressive effects both in vivo and in vitro.^165^ In addition, in various types of thyroid cancer, the expression of CBS is up‐regulated to varying degrees, and the highly expressed CSE can also activate the hedgehog signalling pathway to promote the occurrence and development of thyroid papillary carcinoma.^45^, ^166^

In the adoptive cell transfer mouse model, T cells that overexpressed CSE showed better tumour inhibitory effect than normal T cells, due to the fact that in T cells, the overexpression of CSE does not promote its proliferation and change its phenotype, but rather enhances the inhibition of tumour growth by regulating the concentration of serine, proline, and glycine in the metabolic environment. Inhibiting tumour growth by changing its microenvironment represents a novel approach for tumour immunotherapy.^167^

## 5 METHODS FOR INHIBITING ENDOGENOUS H_2_S

### 5.1 Pharmacological inhibitor

The commonly used inhibitors of endogenous H_2_S are usually inhibitors of H_2_S‐generating enzymes, mainly PAG, BCA, AOAA, HA, and HMPSNE. AOAA was first thought to be a specific inhibitor of CBS. Recent studies have found that AOAA can act as a bidirectional inhibitor of CBS and CSE, possibly due to its inhibitory effect on pyridoxal‐5′‐phosphate, which is a catalytically active cofactor for various enzymes, including CBS and CSE.^168^ PAG is an irreversible and specific inhibitor of CSE, but it also inhibits several transamination reactions in muscle and exhibits some nephrotoxicity, and it cannot cross the blood–brain barrier.^168^ BCA is a reversible CSE inhibitor with broad bioavailability, but it has inevitable neurotoxicity.^169^ HA is a cellular metabolite that can release NO and has antioxidant properties. It can be used as an inhibitor of heme‐containing enzymes including CBS,^170^ but it has an inhibitory effect on CSE at low concentrations.^171^ I157172 is a novel CSE inhibitor that exerts a tumour suppressor effect in BC.^120^ HMPSNE is a newly discovered specific inhibitor of 3‐MST, which acts on the activated cysteine residues in the active site of 3‐MST.^172^ In addition, trifluoroalanine can also inhibit CBS and CSE, and aminoethoxyvinylglycine can inhibit CSE, but these two inhibitors have not been used in cancer research.^171^ In general, AOAA and HA, which are widely used in basic research, have poor targeting, and the side effects of PGA and BCA are relatively large. However, HMPSNE and I157172 are well targeted and have no related side effects reported, which requires further verification of their biological safety. In addition, compounds with aromatic ring‐carbonyl‐S‐pyrimidone structures may be used as new 3‐MST inhibitors.^172^

### 5.2 Target gene sequence

Existing targeted gene silencing approaches including siRNA, shRNA, and CRISPR/Cas9 effectively inhibit endogenous H_2_S production,^126^, ^135^ and all have significant effects. Although 3‐MST‐targeted knockdown has been shown to attenuate cellular biology energetics,^95^ this approach has not been directly applied to cancer research. When compared with pharmacological inhibitors, targeted gene sequences have better specificity, but most researchers still prefer pharmacological inhibition due to the need for expertise and related equipment.^171^

### 5.3 MicroRNA and transcription factors

MicroRNA (miR) and transcription factors can also act as direct regulators of H_2_S synthases. MiR‐24‐3p, miR‐203, and miR‐376a can target CBS to induce apoptosis, and miR‐559 can also target CBS to inhibit cell proliferation.^130^, ^138^, ^173^, ^174^ MiR‐30 family can induce oxidative stress by inhibiting CSE,^175^, ^176^ while miR‐216a also directly targeted CSE to inhibit its expression.^177^ And miR‐4137 can inhibit both CBS and CSE in BC to yield a tumour‐suppressing effect.^114^

In addition, transcription factor Sp1 can increase the expression of CSE, and Nrf2 can activate the ARE upstream of CBS to up‐regulate its expression.^129^, ^156^ Moreover, many molecules can regulate the expression of H_2_S synthases, such as miR‐106a and rpL3, but they cannot directly target these three enzymes and may require other molecules as intermediate bridges to function^164^, ^178^ (Table 1).

**TABLE 1:** Targeted ways to inhibit H_2_S synthases.

Type	Appellation	Target(s)	Refs.
Pharmacological inhibitors	PAG	CSE	^168^
	BCA	CSE	^169^
	AOAA	CSE, CBS	^168^
	HA	CSE, CBS	^170^
	HMPSNE	3‐MST	^172^
	I157172	CSE	^120^
	Trifluoroalanine	CSE, CBS	^171^
	Aminoethoxyvinylglycine	CSE	^171^
Target gene sequence	siRNA	CSE, CBS	^126^
	shRNA	CSE, CBS	^117^, ^146^
	CRISPR/Cas9	CSE, CBS	^125^, ^135^
MicroRNA	MiR‐24‐3p	CBS	^130^
	MiR‐203	CBS	^173^
	MiR‐376a	CBS	^174^
	MiR‐559	CBS	^138^
	MiR‐30	CSE	^175^, ^176^
	MiR‐216a	CSE	^177^
	MiR‐4137	CSE, CBS	^114^
Transcription factor	SP1	CSE	^129^

Abbreviations: 3‐MST, 3‐mercaptopyruvate sulfurtransferase; AOAA, aminooxyacetic acid; BCA, β‐cyano‐L‐alanine; CBS, cystathionine β‐synthase; CSE, cystathionine γ‐lyase; H_2_S, hydrogen sulphide; HA, hydroxylamine; HMPSNE, 2‐[(4‐hydroxy‐6‐methylpyrimidin‐2‐yl)sulfanyl]‐1‐ (naphthalen‐1‐yl)ethan‐1‐one; PAG, DL‐Propargylglycine; SP1, specificity protein 1.

## 6 DISCUSSION

The role of H_2_S in cancer has been increasingly elaborated, and the different roles of endogenous and exogenous H_2_S in cancer provide two approaches for cancer treatment: inhibition of endogenous H_2_S or supplementation of exogenous H_2_S. Although some papers have shown that high expression of H_2_S synthases can promote tumours, this may be due to the high level of H_2_S in the tumour environment (digestive system). In general, inhibiting the synthesis of endogenous H_2_S can inhibit the occurrence and development of cancer. The existing methods of inhibiting endogenous H_2_S include (1), pharmacological inhibitors, (2), siRNA, shRNA, CRISPR/Cas9, (3), miR, and transcription factors. However, pharmacological inhibitors have poor targeting, and their way of action may not only inhibit the production of endogenous H_2_S, and some inhibitors have relatively large side effects. siRNA, shRNA, and CRISPR/Cas9 cannot be applied to human experiments, only to verify the effect of the H_2_S synthases through cell or animal experiments. The same problem exists with miRs and transcription factors. Therefore, in addition to elucidating the deep mechanism of endogenous H_2_S in cancer, the next goal is to develop better‐targeted endogenous H_2_S inhibitors with fewer side effects or endogenous H_2_S inhibition methods that can be applied to humans. Furthermore, in addition to the endogenous H_2_S in tumour cells themselves, H_2_S in T cells and HSCs, for example, can also exert tumour‐suppressive effects by regulating the microenvironment.

In conclusion, although there are various difficulties and challenges, the inhibition of endogenous H_2_S production is a potential cancer treatment.

## AUTHOR CONTRIBUTIONS

Dong‐Dong Wu, Chang‐Yong Yang, and Xin‐Ying Ji conceived the study and drafted the article. Hao‐Jie Chen, Ke Li, Yang‐Zhe Qin, Jing‐Jing Zhou, Tao Li, and Lei Qian, prepared the figures. All authors read and approved the final article.

## FUNDING INFORMATION

This work was supported by grants from the National Natural Science Foundation of China (No. 81802718), the Training Program for Young Backbone Teachers of Institutions of Higher Learning in Henan Province, China (No. 2020GGJS038), the Natural Science Foundation of Education Department of Henan Province, China (No. 21A310003), and the Foundation of Science & Technology Department of Henan Province, China (Nos. 222102310490, 222102310495).

## CONFLICT OF INTEREST STATEMENT

The authors declare that they have no competing interests.

## Data Availability

Not applicable.
